# Assessment of Sleep, Cognitive Performance, and Circulating Cytokines in Adults with Chronic Rhinosinusitis

**DOI:** 10.3390/diagnostics16162671

**Published:** 2026-08-21

**Authors:** Rachel Nolte, Donyea L. Moore, Yitong Huang, Shreya Maharana, Bichun Ouyang, Saloni Sharma, Baharan Fekry, Evan A. Patel, Pete Batra, Mahboobeh Mahdavinia

**Affiliations:** 1 Division of Allergy and Immunology, Department of Internal Medicine, UT Health Houston, 6410 Fannin St, Houston, TX 77030, USA; rachelanolte@gmail.com (R.N.); donyea.moore@gmail.com (D.L.M.); saloni.sharma@uth.tmc.edu (S.S.); 2 Department of Internal Medicine, Dell Medical School, University of Texas at Austin, Austin, TX 78712, USA; 3 Division of Allergy and Immunology, Department of Internal Medicine, Rush University Medical Center, 1725 W Harrison St, Chicago, IL 60612, USA; shreyamaharana8@gmail.com; 4 Department of Mathematical Sciences, Smith College, 10 Elm St, Northampton, MA 01063, USA; yhuang86@smith.edu; 5 Department of Neurological Sciences, Rush University Medical Center, 600 S Paulina St, Chicago, IL 60612, USA; bichun_ouyang@rush.edu; 6 Department of Otorhinolaryngology—Head and Neck Surgery, Rush University Medical Center, 1611 W Harrison St, Chicago, IL 60612, USA

**Keywords:** chronic rhinosinusitis, cognitive function, inflammation, sleep dysfunction, cytokines, IL-6, IL-13

## Abstract

**Background/Objectives:** Chronic rhinosinusitis (CRS) is commonly accompanied by sleep disturbance and cognitive complaints, but the relationships among CRS, cognition, subjective sleep, and circulating inflammatory cytokines remain incompletely understood. **Methods:** In this case–control study, 80 adults with CRS and 98 controls completed validated sleep and sinonasal questionnaires and a 12-test cognitive battery assessing six domains. Plasma cytokines were measured in 101 participants with available biospecimens. Multivariable linear regression and exploratory correlation analyses were performed with false discovery rate correction. **Results:** Adjusted cognitive domain scores did not differ between CRS and controls. In age-by-group interaction models, CRS showed a nominally steeper age-associated decrease in episodic memory (β = −0.12, 95% CI −0.21 to −0.03; *p* = 0.009; q = 0.053), with weaker nominal patterns for working memory (β = −0.10, 95% CI −0.20 to −0.003; *p* = 0.044; q = 0.096) and visuospatial ability (β = −0.08, 95% CI −0.15 to −0.001; *p* = 0.048; q = 0.096). Plasma IL-6 was nominally higher in CRS after adjustment (β = 0.36, 95% CI 0.04 to 0.67; *p* = 0.026; q = 0.18) but did not remain significant after cytokine-level correction. Within CRS, exploratory analyses showed nominal inverse correlations between IL-6 and visuospatial ability (ρ = −0.36; *p* = 0.008; q = 0.236), as well as a nominal positive correlation between IL-13 and semantic memory (ρ = 0.33; *p* = 0.020; q = 0.236); however, none survived correction for multiple comparisons. **Conclusions:** Adults with CRS reported greater sleep-related burden and showed a nominally steeper age-related decrease in episodic memory. IL-6 and IL-13 showed nominal cognitive associations that did not survive correction, supporting the need for larger longitudinal studies to clarify clinical relevance.

## 1. Introduction

Chronic rhinosinusitis (CRS) is a complex inflammatory condition characterized by persistent inflammation of the sinonasal mucosa lasting for more than twelve weeks [1,2]. It affects approximately 4.5 to 12% of the population [2] and is associated with significant impairment in both quality of life and sleep in affected individuals [3]. Beyond sleep disruption, which is a common symptom, the consequences of poor sleep quality extend to various aspects of health, including cognitive function, metabolic disorders, and obesity [4].

Despite the well-established links between sleep disturbances and these health outcomes, the specific impact of sleep quality and other related factors on individuals with CRS has not been thoroughly studied and may not solely rely on sleep disturbance. Cognitive impairment is commonly reported in CRS patients, with declines observed in memory, attention, and executive function [5]. However, the mechanisms underlying these cognitive deficits in the context of CRS remain poorly understood.

Cytokines influence cognition through both central and peripheral mechanisms [6]. On a cellular level, peripheral cytokines can cross the blood–brain barrier or activate the vagus nerve (afferents), signaling and triggering inflammatory responses in the brain that disrupt neuronal function and synaptic plasticity. Specifically, cytokines interact with dopaminergic and cholinergic pathways, which are critical for cognitive processes such as attention, memory, and decision-making [6]. Persistent inflammation is also thought to increase the likelihood of age-related cognitive deterioration over time, further linking inflammatory conditions with neurocognitive decline [7,8].

A growing body of research suggests that sleep disruption, which is common in CRS, is associated with altered immune function and low-grade systemic inflammation. In healthy individuals, sleep fragmentation and sleep deprivation have been linked to changes in circulating pro-inflammatory cytokines, including IL-6 and TNF-α [9,10,11]. Because sustained inflammation has also been associated with cognitive decline, these findings provide a rationale for examining sleep, inflammatory cytokines, and cognitive performance together in CRS.

Given these associations, the present case–control study examined relationships among CRS, cognitive performance, subjective sleep measures, and circulating inflammatory cytokines in adults. We assessed cognitive performance across multiple domains in CRS and control participants, evaluated whether age-related cognitive patterns differed by group, and explored cytokine–cognition associations within the CRS group.

## 2. Materials and Methods

### 2.1. Study Design and Participants

This case–control study assessed and compared cognitive performance and systemic inflammatory biomarkers of adults (≥18 years) with CRS and healthy control participants. The study was approved by the Institutional Review Board at a tertiary care academic medical center in Chicago, IL, and UTHealth Science at Houston, and all participants provided written informed consent prior to enrollment. CRS was diagnosed according to the guidelines of the American Academy of Otolaryngology, which requires the presence of sinonasal symptoms for at least 12 consecutive weeks and positive findings in a non-contrast sinus CT scan or endoscopic evaluation [1]. Participant exclusion criteria included age <18, as well as a known primary sleep disorder, including obstructive sleep apnea (participants with a previously known diagnosis of a primary sleep disorder, including obstructive sleep apnea, identified from medical history at enrollment were excluded). All candidates were screened by the STOP-Bang questionnaire, and only those with scores 0–2 were invited for participation; individuals with pre-existing cognitive impairment, on sedative medication, taking either medications with known impact on cognitive function or oral corticosteroids, or with a cancer diagnosis within the past three years at the time of recruitment and enrollment were excluded due to potential confounding variables. Healthy controls were recruited from the same outpatient population and had no history of CRS or chronic sinonasal symptoms. Asthma and atopy were recorded, but were not exclusion criteria.

### 2.2. Demographic and Clinical Data Collection

Demographic characteristics, comorbid conditions, and medication use were collected using structured questionnaires. All participants were evaluated by an allergy immunologist for comorbid conditions, including allergic rhinitis, asthma, eczema, aspirin-exacerbated respiratory disease, and food allergy. Atopy was defined as a positive skin prick test, in vitro measurement of specific IgE, or a documented clinical history consistent with allergic rhinitis. Asthma diagnosis was confirmed by clinical history and, in some cases, spirometry. Allergic rhinitis and asthma were confirmed by an allergy specialist through obtaining a detailed history and skin testing or in vitro measurement of specific IgE. The presence of nasal polyps was determined by endoscopic examination or by documented medical history. All data were entered into a secure REDCap database.

Medication use was recorded using a structured questionnaire and confirmed by clinician review or by patient electronic medical records.

Data regarding smoking status, depression or anxiety symptoms (e.g., PHQ/GAD), and metabolic disorders (diabetes, hypertension, hyperlipidemia) were not collected and are addressed as limitations.

### 2.3. Subjective Sleep and Cognition Assessments

We used six questionnaires assessing demographics and sleep habits, including (1) the Pittsburgh Sleep Quality Index (PSQI) [12], (2) the Functional Outcomes of Sleep Questionnaire (FOSQ10) [13], (3) the Epworth Sleepiness Scale (ESS) [14], (4) the Munich Chronotype Questionnaire (MCTQ) [15], (5) the Sinonasal Outcome Test (SNOT-22) [16], (6) and the Chronic Sinusitis Survey (see Supplementary Materials) [17]. Additionally, a cognitive assessment consisting of twelve subtests was conducted to evaluate six cognitive domains, including Episodic Memory, Attention/Processing Speed, Visuospatial Abilities, Semantic Memory, Working Memory, and Executive Function [18]. Immediate and delayed word-list recall were used to assess episodic memory [19]; Attention/processing speed was assessed using Digit Symbol Substitution [20] and Number Comparison [21]; visuospatial ability was assessed using Raven’s Progressive Matrices [22] and Line Orientation; semantic memory was assessed using the Boston Naming Test [23], verbal/phonemic fluency [24] and category/semantic fluency [25]; working memory was assessed using Digit Span tasks [20]; and executive function was assessed using Stroop Color and Word tasks [26]; more details are provided in Supplementary Materials. Cognitive domain scores were calculated as the sum of raw subtest scores within each domain. All the above-described tests in the cognitive assessment were quantified, and the significant differences were analyzed between the CRS and control patients.

Venous blood samples were collected from the study participants following standardized venipuncture procedures during routine daytime clinic hours (between 9:00 AM and 5:00 PM). Blood was collected into sodium heparin tubes, centrifuged at 2150 rpm for 15 min, and plasma was aliquoted and stored at −80 °C until analysis. Plasma concentrations of interleukin-6 (IL-6), IL-8, IL-10, IL-12-p70, IL-13, IFN-γ, and TNF-α were quantified using Meso scale Diagnostics (MSD) U-PLEX multiplex electrochemiluminescence assay (MSD; Catalog #K15067L-2). Assays were performed per the manufacturer’s instructions. Samples were run in duplicate, and laboratory personnel were blinded to participants’ CRS status, as samples were de-identified and assigned unique IDs. CRS and control samples were randomized across assay plates to minimize batch effects.

### 2.4. Statistical Analysis

Demographic characteristics, clinical measures, and survey-based outcomes related to cytokines, cognition, and sleep quality were summarized using means and standard deviations for continuous variables, and frequency distributions for categorical variables. Group differences between healthy controls and individuals with CRS were assessed using the Wilcoxon rank-sum test for non-normally distributed continuous variables and the chi-squared test or Fisher’s exact test for categorical variables. For all descriptive groupwise comparisons, raw (unadjusted) *p*-values were provided, and we applied the threshold of *p* < 0.05 for statistical significance throughout. Group differences in cognitive performance, cytokine concentrations, and sleep variables were assessed using two complementary approaches. First, unadjusted Wilcoxon rank-sum test provided descriptive between-group comparisons. Second, multiple linear regression provided adjusted comparisons controlling for age, sex, race, BMI, and years of education. For cytokine analysis specifically, regression was performed on log-transformed cytokine values to approximate a Gaussian distribution. Analyses were conducted using available complete cases for each model; no imputation was performed. Benjamini–Hochberg false discovery rate correction was applied within predefined families of related tests, and both nominal *p*-values and FDR q-values were reported. The 42 exploratory comorbidity subgroup comparisons were treated as one analysis family and adjusted using the Benjamini–Hochberg procedure; both nominal *p*-values and FDR-adjusted q-values are reported.

Effect sizes by Cohen’s d were reported alongside all primary statistical tests. In secondary analyses examining whether age-related cognitive performance differs between groups, regression models with demographic covariates were further adjusted to include the age x group interaction term. In these models, the age-by-group interaction coefficient represented the difference in age-associated cognitive slopes between CRS and control participants. For each model, the total R^2^ was reported to indicate the proportion of variance in the cognitive outcome explained by all predictors combined. Partial R^2^ was also reported for the interaction term to indicate the proportion of variance attributable to differential age effects between CRS and controls, after accounting for all other covariates.

Finally, exploratory analyses were conducted to characterize associations between inflammation, sleep, and cognitive performance within the CRS group. Both bivariate Spearman correlations and partial Spearman correlations adjusting for age, sex, BMI, years of education, atopy, asthma, and nasal polyp status were computed. The Benjamini–Hochberg procedure was applied separately to control the false discovery rate. Statistical analyses were conducted using R version 4.4.0.

### 2.5. Use of Generative AI Tools

Generative AI was not used to generate the original manuscript or its scientific content.

## 3. Results

### 3.1. Demographics and Clinical Characteristics

A total of 178 participants were enrolled in the study, including 80 individuals with CRS and 98 control participants. The demographic characteristics of the participants are summarized in Table 1. Significant differences were observed in age (*p* = 0.01) and sex distribution (*p* = 0.02) between the two groups, while all other demographic characteristics, including BMI, race, ethnicity, and years of education, were not significantly different between groups (Table 1). Within the CRS cohort, 56.3% had asthma, 78.8% were classified as atopic, and 48.8% had nasal polyps (Table 1).

### 3.2. Cognitive Function Profile

Cognitive domain scores were first compared between CRS and control participants. Cognitive scores did not differ significantly between the CRS and control groups in unadjusted Wilcoxon rank-sum tests (*p* > 0.1) or in multiple linear regression models adjusting for demographic covariates (all *p* > 0.1; Table 2). After adjustment, the processing-speed difference was reduced, while the semantic-memory estimate changed little; neither difference was statistically significant.

Group means and standard deviation (SD) are unadjusted sample statistics; adjusted group mean differences and 95% confidence interval (CI) are derived from linear regression models adjusting for age, sex, race, BMI, and years of education as covariates. False discovery rates (FDR) were obtained using the Benjamini–Hochberg correction (BH) across the six domains.

Given that age is a well-established confounding factor in cognitive decline, we investigated whether the association between age and cognitive performance differs between CRS and controls, using a multivariate regression model with an interaction term between age and group. Sex, race, BMI, and years of education were included as covariates to account for demographic confounders. The multivariable models explained 21% to 32% of the variance in the cognitive performance across six domains (Table 3). The age × group interaction term explained 4.1% of the variance in episodic memory, 2.4% in working memory, and 2.3% in visuospatial abilities. The interaction term did not reach the statistical significance level of 0.05 after BH correction across the six cognitive domains (Table 3).

**Figure 1:**
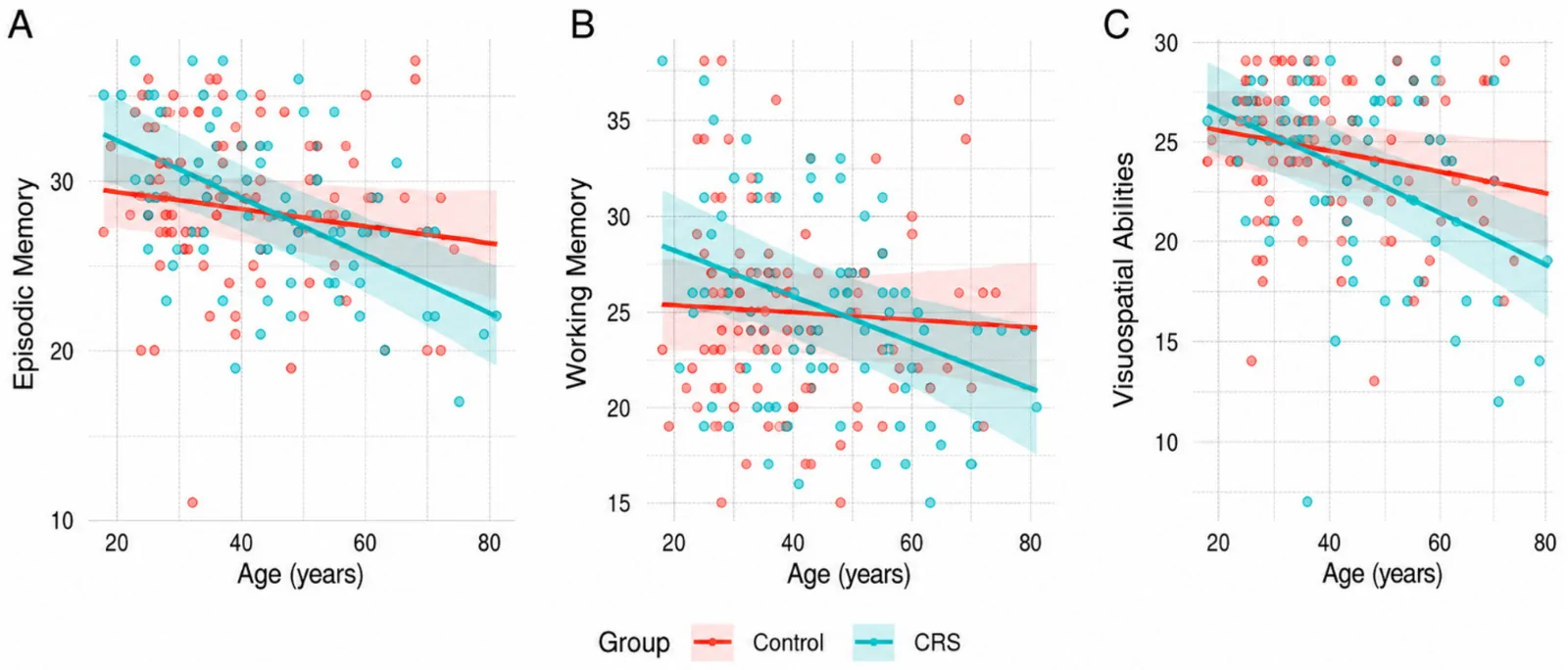
**Differential age effect on cognitive performance between CRS and control subjects**. Individual observations of cognitive assessment scores are plotted against age as points (*N* = 178, including 98 controls in red and 80 CRS subjects in blue). Model-predicted values (lines) and 95% confidence intervals (shaded regions) for (**A**) episodic memory, (**B**) working memory, and (**C**) visuospatial abilities, plotted as a function of age and group. Predictions were derived from linear models regressing each cognitive outcome on age, group, their interaction, and covariates (sex, race, BMI, and years of education).

Nevertheless, nominally steeper age-associated decreases were observed in CRS for episodic memory (*p* = 0.009, q = 0.053), working memory (*p* = 0.044, q = 0.096), and visuospatial abilities (*p* = 0.048, q = 0.096), although these did not survive FDR correction. Full covariate-level regression outputs for the age-by-group interaction models are provided in Supplementary Table S3, and the corresponding age-associated cognitive patterns are shown in Figure 1 and Supplementary Figure S1.

### 3.3. Cytokines

Cytokine analyses included 101 participants with available plasma samples (53 with CRS and 48 controls). In unadjusted comparisons, CRS participants showed moderately higher circulating IL-6 (Cohen’s d = 0.59; Wilcoxon rank-sum *p* = 0.005) and IL-8 (Cohen’s d = 0.49; Wilcoxon rank-sum *p* = 0.02) concentrations than controls, suggesting nominal group differences in circulating inflammatory cytokine levels. After adjustment for age, sex, race, BMI, and years of education, IL-6 remained nominally higher in CRS participants (β = 0.36; *p* = 0.026), although no individual cytokine difference survived Benjamini–Hochberg FDR correction (all FDR > 0.05). IL-10, IL-12-p70, IL-13, IFN-gamma, and TNF-alpha did not differ between groups in either unadjusted or adjusted analyses. Cytokine concentrations are presented in Table 4.

Within the CRS group, no significant association was found between any of the systemic inflammatory markers and atopy, asthma, and nasal polyps.

Cytokine values are reported as mean (SD) for raw values. Cohen’s d was calculated as the standardized mean difference on log-transformed cytokine concentrations. Group differences were assessed using linear regression on log-transformed cytokine values, controlling for age, sex, BMI, race, and years of education. Adjusted regression coefficients (adjusted beta) represent the difference in log cytokine between CRS and controls and are presented with 95% confidence intervals. Benjamini–Hochberg FDR correction was applied across the seven cytokines.

### 3.4. Subjective Sleep Assessments

In bivariate analysis of sleep variables, CRS participants reported worse subjective sleep measures than controls (Table 5). These differences remained significant in multivariable models adjusted for age, sex, race, BMI, and years of education. Higher PSQI global scores indicate poorer subjective sleep quality; CRS participants had higher PSQI scores than controls after adjustment (β = 3.78, 95% CI 2.53 to 5.04; *p* < 0.001; FDR q < 0.001). Higher ESS scores indicate greater daytime sleepiness; CRS participants also had higher ESS scores after adjustment (β = 2.87, 95% CI 1.52 to 4.22; *p* < 0.001; FDR q < 0.001). Lower FOSQ composite scores indicate poorer sleep-related functioning; CRS participants had lower FOSQ scores after adjustment (β = −4.41, 95% CI −6.39 to −2.44; *p* < 0.001; FDR q < 0.001). Within the CRS group, PSQI, ESS, and FOSQ scores did not differ significantly by asthma, atopy, or nasal polyp status (all *p* ≥ 0.12).

Although these survey instruments assess different aspects of sleep and fatigue, significant correlations were observed between each set of PSQI, ESS, and FOSQ scores (*p* < 0.001 for every set of comparisons adjusted for multiple comparisons).

Additional sleep-related measures are shown in Supplementary Figure S2. Compared with controls, CRS participants showed poorer sleep-related functioning, longer reported sleep latency measures, greater SNOT-22 sleep-domain burden, greater daytime sleepiness, and poorer global sleep quality (all *p* < 0.001).

### 3.5. Associations Between Cognition, Inflammation, and Sleep in Participants with CRS

To examine within-CRS patterns, exploratory bivariate Spearman correlation analyses were conducted between the six cognitive domains and circulating inflammatory markers. Across the 42 bivariate cytokine–cognition correlations, none remained statistically significant after Benjamini–Hochberg FDR correction. Four associations showed nominal significance (unadjusted *p* < 0.05; all *q* = 0.236) and are considered exploratory. Covariate-adjusted partial correlations showed directionally similar patterns, but none remained significant after FDR correction.

Several nominal correlations were observed: IL-6 was inversely correlated with visuospatial ability (ρ = −0.36; *p* = 0.008; FDR q = 0.236), IL-8 was inversely correlated with processing speed (ρ = −0.34; *p* = 0.013; FDR q = 0.236), IL-10 was positively correlated with executive function (ρ = 0.33; *p* = 0.017; FDR q = 0.236), and IL-13 was positively correlated with semantic memory (ρ = 0.33; *p* = 0.020; FDR q = 0.236).

Partial Spearman correlation analyses adjusting for age, sex, BMI, years of education, asthma, atopy, and nasal polyp status showed similar directional patterns. IL-13 remained positively correlated with semantic memory scores (partial ρ = 0.40), while IL-6 showed inverse correlations with visuospatial ability (partial ρ = −0.24) and executive function (partial ρ = −0.32). However, none of these partial correlations remained significant after Benjamini–Hochberg correction. These findings are shown in Supplementary Figure S3.

Similarly, subjective sleep measures were not significantly correlated with cognitive performance in CRS participants in either bivariate Spearman analyses or adjusted partial Spearman analyses. These sleep–cognition findings are shown in Supplementary Figure S4.

We also found that, within CRS participants, total sleep scores were not significantly associated with circulating cytokine levels after FDR correction. Supplementary Figure S5.

### 3.6. Exploratory CRS-Related Comorbidity Subgroup Analyses

Across the 42 exploratory subgroup comparisons, none remained significant after FDR correction (all q ≥ 0.171).

Atopy: Non-atopic CRS participants had nominally lower episodic memory (*p* = 0.004; q = 0.171) and composite cognition scores (*p* = 0.043; q = 0.467) than non-atopic controls; neither difference survived FDR correction. No nominal cognitive differences were observed within the atopic stratum.

Asthma: No nominal cognitive differences were observed between CRS and control participants within either asthma stratum.

Nasal polyps: CRS without nasal polyps (CRSsNP) participants had nominally lower semantic memory (*p* = 0.033; q = 0.467) and composite cognition scores (*p* = 0.044; q = 0.467) than controls; neither difference survived FDR correction. CRS with nasal polyps (CRSwNP) had comparable cognitive performance.

## 4. Discussion

This case–control study examined associations among CRS, systemic inflammatory cytokines, subjective sleep burden, and cognitive performance. Adults with CRS reported worse subjective sleep outcomes and showed a possible age-associated pattern of lower cognitive performance in selected domains. The strongest cognitive signal was observed for episodic memory, which approached FDR-adjusted significance, with weaker nominal patterns in working memory and visuospatial abilities. CRS participants also showed numerically higher plasma IL-6 levels, although this association did not remain significant after correction across cytokines.

Age-related analysis suggested that rather than being related to global cognitive impairment in all adults, CRS may be related to increased vulnerability to cognitive decline with increasing age of the patient. This pattern was most robust for episodic memory, with less robust associations for working memory and visuospatial ability.

These findings are consistent with the broader concept that chronic low-grade inflammation may be associated with cognitive vulnerability during aging [7,8]. In the present cross-sectional study, CRS showed nominal age-associated cognitive patterns, most notably in episodic memory, and IL-6 showed nominal associations with selected cognitive outcomes, consistent with the prior literature linking IL-6 to brain health and cognition [27]. These findings are also consistent with our prior actigraphy-based work in CRS, which suggested links between circadian disruption and age-related memory patterns [28]. However, these findings did not survive FDR correction and should be interpreted as exploratory.

Exploratory analyses identified nominal correlations between selected inflammatory markers and cognitive performance in CRS patients. IL-6 was inversely correlated with visuospatial ability, and IL-8 was inversely correlated with processing speed in bivariate analyses; however, these associations did not survive FDR correction. Adjusted partial Spearman analyses showed similar directional patterns but also did not remain significant after correction.

Conversely, IL-13 showed a nominal positive association with semantic memory in adults with CRS, although this association did not survive correction for multiple comparisons. In exploratory subgroup analyses, CRSsNP participants had nominally lower semantic memory and composite cognition scores than controls, whereas participants with CRSwNP had comparable cognitive performance. None of these comparisons survived FDR correction.

IL-10 showed a nominal positive correlation with executive function. This finding is consistent with experimental evidence suggesting that IL-10 may limit neuroinflammation and support brain repair after injury [29]. However, because this association did not survive multiple-comparison correction, it should be interpreted as hypothesis-generating.

Previous studies, including those conducted by our group, have demonstrated that measures of circadian disruption are associated with cognitive function [28,30,31,32]. Despite significantly lower sleep quality in CRS patients (Supplementary Figure S2), we did not observe an association between subjective sleep measures and cognitive performance. It is noteworthy that our sleep assessments were limited to subjective measures and did not include objective sleep evaluations. Therefore, our results can only indicate that subjective sleep scores were not significantly correlated with cognitive performance in this cohort. However, consistent with prior research [9,10,11], in our cohort, as shown in Supplementary Figure S5, higher PSQI scores were positively associated with IL-6 and IL-8 in unadjusted analyses (IL-6: ρ = 0.23, *p* = 0.0297; IL-8: ρ = 0.31, *p* = 0.0028; *n* = 93). These findings are consistent with prior reports linking poor sleep quality to systemic inflammation [33]. After adjustment for age, sex, race, BMI, and years of education, the IL-6 association was attenuated (partial ρ = 0.19, *p* = 0.073), whereas IL-8 remained nominally associated after adjustment (partial ρ = 0.24, *p* = 0.0222; *n* = 92); however, the association did not survive FDR correction. In CRS-stratified analyses, neither IL-6 nor IL-8 was significantly associated with PSQI among CRS participants (*n* = 44) but did not establish a CRS-specific sleep–inflammation–cognition pathway.

Asthma did not explain the observed cognitive patterns in this cohort, although the study was not powered for definitive subgroup inference.

There are multiple limitations to this study. First, due to the cross-sectional design and relatively small sample size, there was limited power to detect cognitive differences and establish causation. Second, due to the analysis based on only those participants that had biospecimens available, the subset of subjects used for cytokine analyses was smaller. Although descriptive characteristics did not differ significantly between those with and without cytokine data, missingness may not have been completely random and limited adjusted and subgroup analyses (Supplementary Figure S6 and Table S4).

Because OSA was excluded only on the basis of subjective screening (STOP-Bang) or a previously known diagnosis, undiagnosed mild OSA may have remained in either group. OSA can cause sleep fragmentation and intermittent hypoxemia and has measurable systemic and end-organ effects beyond those captured by subjective sleep questionnaires [34,35]. Residual OSA may have played a role in the differences that we noticed in how people felt about their sleep, and it could have mixed things up with the cytokine and cognitive results that we were exploring.

Blood sampling was conducted at routine clinic visit hours between 9:00 AM and 5:00 PM. This broad collection window may have introduced time-of-day variability across cytokine measurements and reduced the precision of the estimated group differences, particularly for IL-6, which is known to exhibit diurnal variation. Moreover, because sleep was assessed only through questionnaires, we may not have captured important aspects of sleep disruption, such as nighttime fragmentation or apnea-related events. This limitation may partly explain why we did not observe an association between sleep and cognition; therefore, the null finding should be interpreted cautiously rather than as evidence that no relationship exists. Future studies should include objective measures, such as actigraphy to characterize sleep–wake patterns, as previously used by our group [28], and polysomnography or home sleep apnea testing when sleep-disordered breathing is suspected.

The information about depression and anxiety levels, smoking status, and metabolic comorbidity was not available. Because these factors may independently influence sleep, systemic inflammation, and cognitive performance, their potential contribution to the observed associations cannot be excluded. Accordingly, our findings should be interpreted as exploratory and hypothesis-generating rather than as evidence of causal or mechanistic relationships.

## 5. Conclusions

These findings suggest that adults with chronic rhinosinusitis may experience a greater sleep-related burden and may be more susceptible to age-associated changes in specific cognitive domains, particularly episodic memory. Although the cytokine analyses were exploratory and did not retain statistical significance after correction for multiple comparisons, the observed patterns may reflect inflammatory mechanisms that warrant further investigation in the context of CRS-related cognitive aging.

## Figures and Tables

**Table 1:** Demographic and Clinical Characteristics of a cohort of 178 patients with or without CRS.

Demographic and Clinical Characteristic Variables	Control (*n* = 98)	CRS (*n* = 80)	*p*-Value *
Age (Mean ± SD)	39.53 ± 14.02	45.25 ± 14.72	0.01
BMI (Mean ± SD)	28.53 ± 6.67	29.92 ± 6.24	0.16
Race, *n* (%)			0.09
American Indian	0	1 (1.25%)	
Asian	11 (11.2%)	3 (3.75%)	
Native Hawaiian	0	0	
African American	27 (27.6%)	18 (22.5%)	
White	38 (38.8%)	45 (56.25%)	
More than one race	2 (2.04%)	0	
Latino	18 (18.4%)	13 (16.25%)	
Unknown	2 (2.04%)	0	
Ethnicity, *n* (%)			0.97
Hispanic or Latino	23 (23.47%)	19 (23.75%)	
Not Hispanic or Latino	75 (76.53%)	61 (76.25%)	
Sex, Female, *n* (%)	79 (80.61%)	51 (63.75%)	0.02
Years of Education (Mean ± SD)	16.63 ± 2.67	15.87 ± 2.79	0.07
Asthma, *n* (%)	15 (15.3%)	45 (56.3%)	<0.001
Atopy, *n* (%)	29 (29.6%)	63 (78.8%)	<0.001
Polyp, *n* (%)	0(0%)	39 (48.8%)	<0.001

* Descriptive analysis with unadjusted *p*-values. Level of significance at *p* < 0.05.

**Table 2:** Characteristics of cognitive performance.

Domain	Control Mean (SD)	CRS Mean (SD)	d	Adjusted β (95% CI)	*p*; FDR
Episodic memory	28.92 (4.70)	28.28 (4.61)	−0.14	0.36 (−1.01, 1.73)	0.61; 0.80
Working memory	24.92 (5.03)	24.73 (5.28)	−0.04	0.60 (−0.90, 2.10)	0.43; 0.80
Visuospatial abilities	24.42 (3.61)	23.63 (4.56)	−0.20	−0.72 (−1.86, 0.42)	0.22; 0.65
Semantic memory	56.67 (9.93)	53.98 (8.92)	−0.28	−2.2 (−5.01, 0.65)	0.13; 0.65
Processing speed	86.97 (14.57)	83.05 (13.53)	−0.28	−0.62 (−4.51, 3.26)	0.75; 0.80
Executive function	88.11 (17.38)	85.30 (15.94)	−0.17	−0.63 (−5.51, 4.25)	0.80; 0.80

Adjusted for age, sex, race, BMI, and years of education. d: Cohen’s d.

**Table 3:** The age × group interaction effects on cognitive performance across six domains.

Outcome	β Age × Group	95% CI	*p*; FDR	Partial R^2^	Model R^2^
Episodic memory	−0.12	−0.21, −0.03	0.01; 0.053	0.041	0.26
Working memory	−0.10	−0.20, −0.003	0.04; 0.096	0.024	0.23
Visuospatial abilities	−0.08	−0.15, −0.001	0.05; 0.096	0.023	0.31
Processing speed	−0.01	−0.27, 0.25	0.93; 0.93	0.00	0.32
Executive function	0.02	−0.30, 0.35	0.89; 0.93	0.001	0.24
Semantic memory	−0.05	−0.23, 0.14	0.61; 0.92	0.001	0.22

β coefficients represent the difference in age slopes between the CRS group and controls (the reference group) for cognitive scores. FDR values are Benjamini–Hochberg false discovery rate. Partial R^2^ indicates the proportion of variance in each outcome uniquely explained by the term; model R^2^ indicates the total variance explained by all predictors combined. All models include age, group, their interaction, sex, race, BMI, and years of education.

**Table 4:** Inflammatory markers of a cohort of participants with or without CRS.

Cytokine	Control, Mean (SD) *n* = 48	CRS, Mean (SD) *n* = 53	d	β, CRS vs. Control (95% CI)	*p* Value *	FDR *
IL-6	0.94 (0.69)	1.79 (2.21)	0.59	0.36 (0.04, 0.67)	0.03	0.18
IL-8	9.30 (4.59)	13.89 (16.17)	0.49	0.20 (−0.02, 0.43)	0.08	0.19
IL-10	0.30 (0.22)	0.61 (1.11)	0.41	0.31 (−0.04, 0.37)	0.08	0.19
IL-12p70	0.34 (0.22)	0.34 (0.20)	0.07	0.09 (−0.19, 0.37)	0.53	0.74
IL-13	1.44 (0.93)	1.51 (0.85)	0.05	0.21 (−0.06, 0.37)	0.13	0.22
IFN-γ	10.08 (14.60)	31.55 (100.79)	0.20	−0.02 (−0.46, 0.42)	0.92	0.92
TNF-α	1.38 (0.47)	1.46 (0.66)	0.01	−0.05 (−0.24, 0.15)	0.65	0.75

*: Adjusted for age, sex, race, BMI, and years of education. d: Cohen’s d.

**Table 5:** Subjective Sleep variables of a cohort of participants with or without CRS.

Sleep Variable	Control, Mean (SD)	CRS, Mean (SD)	d	β, CRS vs. Control (95% CI)	*p* Value *	FDR *
PSQI global score	5.45 (3.35)	9.19 (3.99)	1.03	3.78 (2.53 to 5.04)	<0.001	<0.001
ESS total score	5.74 (3.70)	8.47 (4.46)	0.66	2.87 (1.52 to 4.22)	<0.001	<0.001
FOSQ composite score	34.01 (6.33)	30.00 (5.02)	−0.69	−4.41 (−6.39 to −2.44)	<0.001	<0.001

*: Adjusted for age, sex, race, BMI, and years of education. d: Cohen’s d.

## Data Availability

The original contributions presented in this study are included in the article/Supplementary Material. Further inquiries can be directed to the corresponding author.

## Supplementary Materials

The following supporting information can be downloaded at https://www.mdpi.com/article/10.3390/diagnostics16162671/s1, Figure S1: Age-associated cognitive performance categorized by CRS status; Figure S2: Characterization of sleep assessments in CRS versus controls; Figure S3. Partial Spearman correlations between inflammatory markers and cognitive assessments in participants with CRS; Figure S4. Partial Spearman correlations between subjective sleep variables and cognitive assessments in participants with CRS; Figure S5. Associations between PSQI global score and IL-6/IL-8; Figure S6. Participant flow and data availability; Table S1. Multivariable linear regression analysis predicting working memory performance; Table S2. Multivariable linear regression analysis predicting episodic memory performance; Table S3. Multivariable regression models for cognitive domain scores; Table S4. Comparison of participants with and without available cytokine data.

## Abbreviations

CRS	Chronic rhinosinusitis
CRSsNP	Chronic rhinosinusitis without nasal polyps
CRSwNP	Chronic rhinosinusitis with nasal polyps
CSS	Chronic Sinusitis Survey
ESS	Epworth Sleepiness Scale
MCTQ	Munich Chronotype Questionnaire
FOSQ10	Functional Outcomes of Sleep Questionnaire
IL-6	Interleukin 6
MSFSC	mid-point of sleep on free days
PSQI	Pittsburgh Sleep Quality Index
SNOT-22	22-item sinonasal outcome test
