# Supplementary material for: Assessment of Sleep, Cognitive Performance, and Circulating Cytokines in Adults with Chronic Rhinosinusitis
Source: Diagnostics (Basel). 2026 Aug 21;16(16):2671. doi: 10.3390/diagnostics16162671 (PMC13512216; doi:10.3390/diagnostics16162671)
Supplement: Supplementary file 1 [file diagnostics-16-02671-s001.zip › diagnostics-4445248-supplementary.pdf]

Supplementary S1

Supplementary Figures

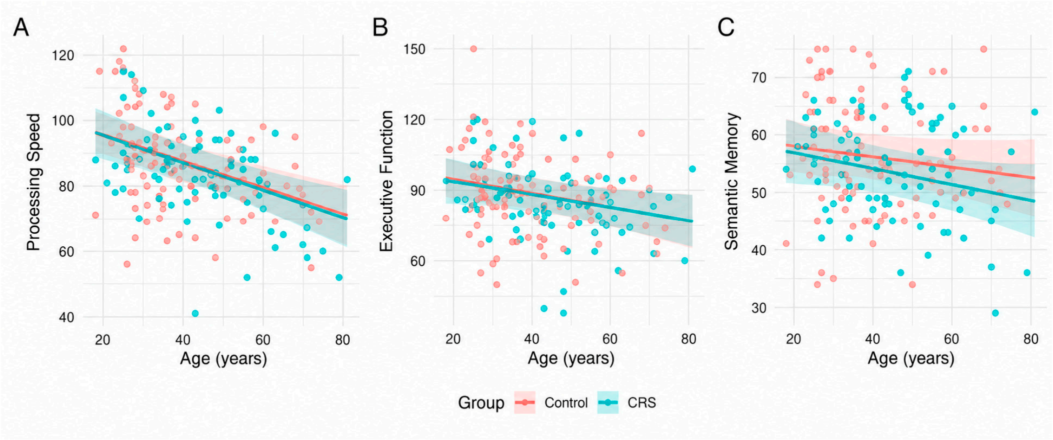

Supplementary Figure S1. Age-associated cognitive performance categorized by CRS status. Individual cognitive domain scores are plotted against age for control and CRS participants. Lines and shaded regions show fitted values and 95% confidence intervals from linear models including age, group, the age-by-group interaction, sex, race, BMI, and years of education. Panels show (A) processing speed, (B) executive function, and (C) semantic memory.

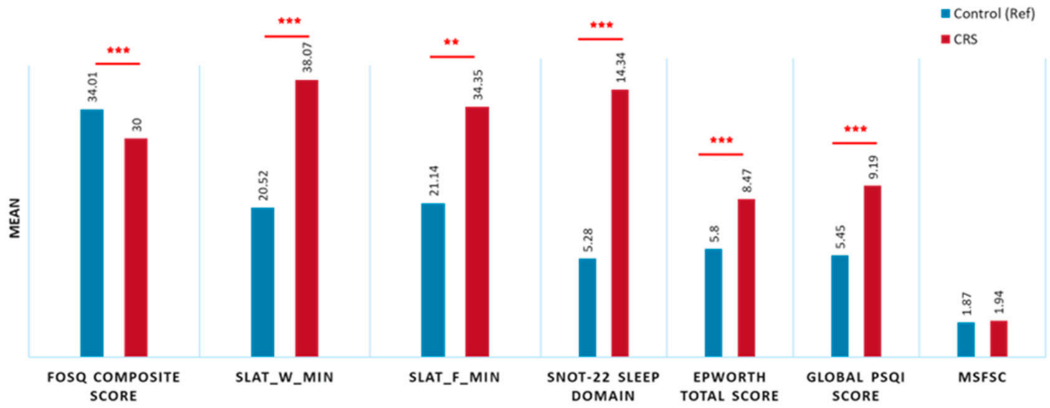

Supplementary Figure S2. Characterization of sleep assessments in CRS versus controls. Mean sleep-related scores are shown for control and CRS participants. For PSQI, ESS, and FOSQ, adjusted group comparisons are reported in the main manuscript. Higher PSQI and ESS indicate poorer sleep quality and greater daytime sleepiness, respectively; lower FOSQ indicates poorer sleep-related functioning.

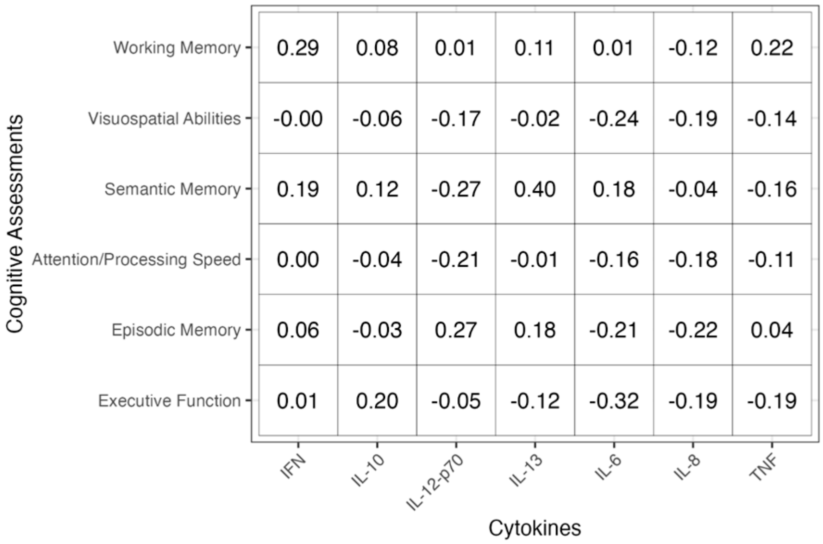

Supplementary Figure S3. Partial Spearman correlations between inflammatory markers and cognitive assessments in participants with CRS. Partial correlation coefficients adjusted for age, sex, BMI, years of education, asthma, atopy, and nasal polyp status are shown in the figure. No correlation remained statistically significant after Benjamini–Hochberg FDR correction.

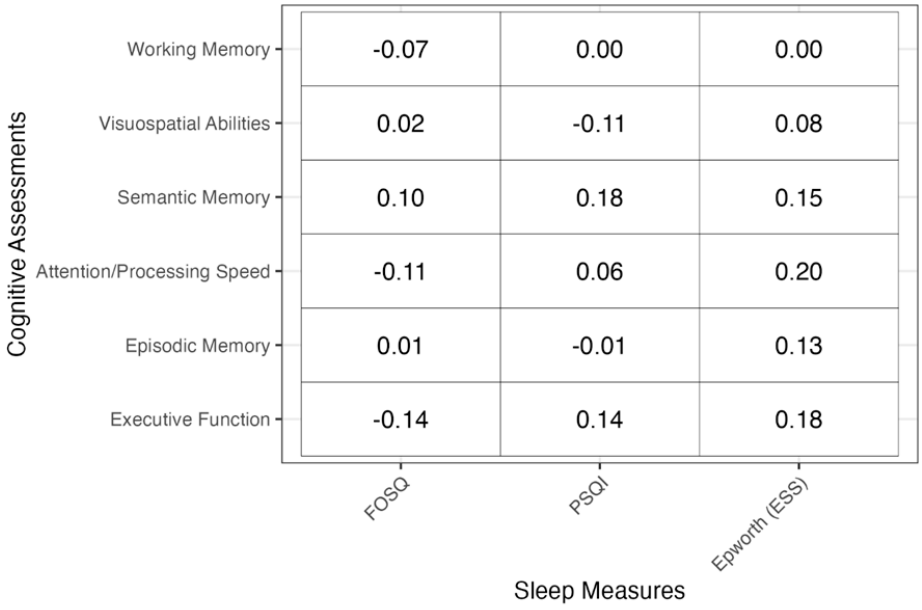

Supplementary Figure S4. Partial Spearman correlations between subjective sleep variables and cognitive assessments in participants with CRS. Partial correlation coefficients were adjusted for age, sex, BMI, years of education, asthma, atopy, and nasal polyps, as shown in the figure. No correlation remained statistically significant after Benjamini–Hochberg FDR correction.

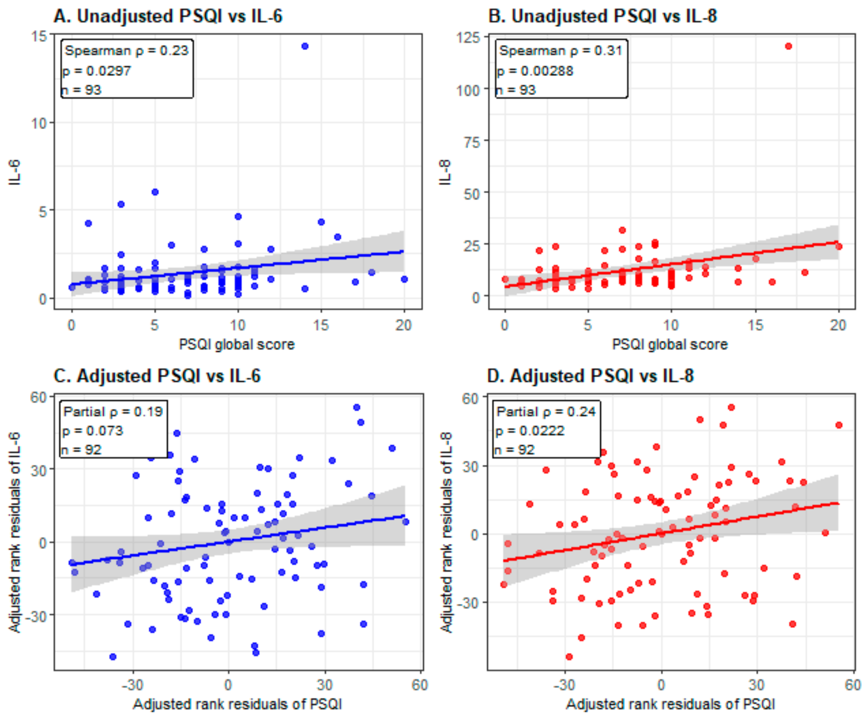

Supplementary Figure S5. Associations between PSQI global score and IL-6/IL-8. (A) Unadjusted Spearman correlation between PSQI global score and IL-6. (B) Unadjusted Spearman correlation between PSQI global score and IL-8. (C) Adjusted partial Spearman correlation between PSQI and IL-6. (D) Adjusted partial Spearman correlation between PSQI and IL-8. Adjusted analyses controlled for age, sex, race, BMI, and years of education. IL-6 is shown in blue and IL-8 in red. Analyses were performed using available complete cases.

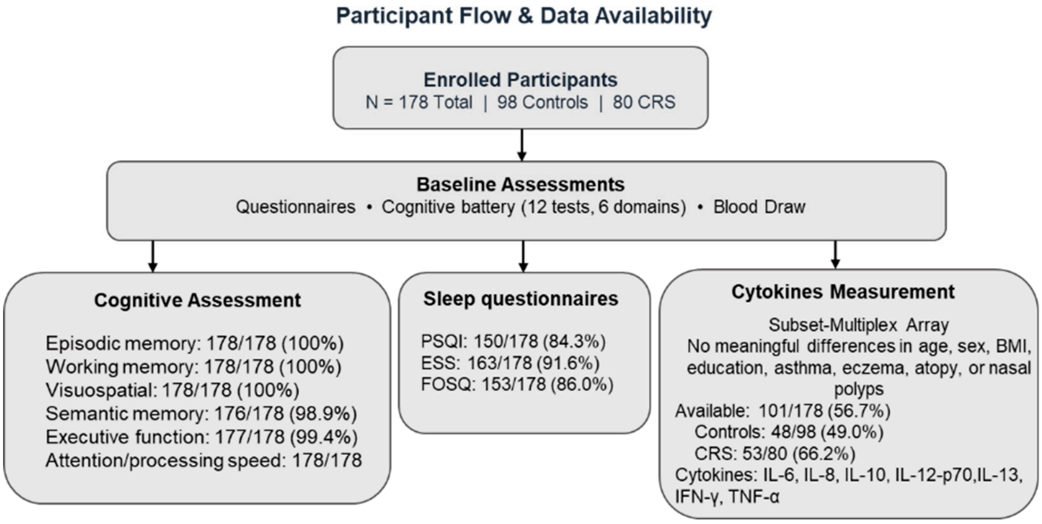

Supplementary Figure S6. Participant flow and data availability. The full cohort included 178 participants, including 98 controls and 80 adults with CRS. Baseline assessments included sleep questionnaires, cognitive testing, and blood collection. Cytokine data were available for 101 participants, including 48 controls and 53 CRS participants.

Supplementary S2

Supplementary Tables

Supplementary Table S1. Multivariable linear regression analysis predicting working memory performance. Coefficients were estimated using the lm function in R.

Predictor	Estimate	Standard Error	t-value	p-value
Intercept	21.31357	3.5911	5.94	<0.001
Age	-0.01984	0.03441	-0.58	0.565
Group^{+}	4.88502	2.24345	2.18	0.031
Sex^{++}	0.94532	0.83756	1.13	0.261
Race^{+++}				
American Indian	-6.72583	4.68761	-1.43	0.1533
Asian	2.35543	1.42765	1.65	0.101
African American	-1.54515	0.9075	-1.7	0.091
More than one race	-0.25141	3.41166	-0.07	0.941
Latino	-3.32076	1.05375	-3.15	0.002
Unknown	4.03510	3.33991	1.21	0.229
BMI	0.00119	0.05877	0.02	0.984
Years of Education	0.28842	0.14291	2.02	0.045
Age × Group	-0.10096	0.0498	-2.03	0.044

Group, sex, and race were treated as categorical variables. Reference categories: control group (+), female (++), and white (+++). The p-values are two-sided.

Supplementary Table S2. Multivariable linear regression analysis predicting episodic memory performance. Coefficients were estimated using the *lm* function in R.

Predictor	Estimate	Standard Error	t-value	p-value
Intercept	23.74466			<0.001
Age	-0.05024	0.03118	-1.61	0.1091
Group+	5.43867	2.03329	2.67	0.0082
Sex++	-0.62459	0.7591	-0.82	0.4118
Race+++<br>American Indian<br>Asian<br>African American<br>More than one race<br>Latino<br>Unknown	 0.52311<br>0.77448<br>-0.47428<br>-2.41478<br>-1.55324<br>-0.8586	 4.24848<br>1.29391<br>0.82249<br>3.09205<br>0.95503<br>3.02703	 0.12<br>0.6<br>-0.58<br>-0.78<br>-1.63<br>-0.28	 0.9022<br>0.5503<br>0.565<br>0.436<br>0.1058<br>0.777
BMI	-0.00984	0.05326	-0.18	0.8537
Years of Education	0.47816	0.12953	3.69	0.0003
Age × Group	-0.11969	0.04514	-2.65	0.0088

Group (CRS versus control), sex, and race were treated as categorical variables. Reference categories: control group (+), female (++), and white (+++). The p-values are two-sided.

Supplementary Table S3. Multivariable regression models for cognitive domain scores.

Term	Coefficient	Standard Error	95% CI	p-value	FDR
**Processing Speed**					
Age × Group	-0.01	0.13	-0.27, 0.25	0.93	0.93
Group, CRS	-0.12	5.88	-11.70, 11.50	0.98	0.98
Age	-0.40	0.09	-0.58, -0.23	<0.01	<0.01
BMI	-0.33	0.15	-0.63, -0.02	0.04	0.10
Years of Education	0.74	0.38	0.00, 1.48	0.05	0.06
Sex, Male	-0.80	2.20	-5.14, 3.54	0.72	0.72
African American	-6.13	2.38	-10.80, -1.43	0.01	0.03
Latino	-4.06	2.76	-9.52, 1.39	0.14	0.14
Asian	0.83	3.74	-6.56, 8.22	0.83	0.83
**Executive Function**					
Age × Group	0.02	0.16	-0.30, 0.35	0.89	0.93
Group, CRS	-1.58	7.38	-16.20, 13.00	0.83	0.98

Age	-0.30	0.11	-0.52, -0.07	0.01	0.03
BMI	-0.71	0.19	-1.09, -0.33	<0.01	<0.01
Years of Education	0.70	0.47	-0.23, 1.63	0.14	0.14
Sex, Male	3.46	2.75	-1.98, 8.90	0.21	0.49
African American	-5.19	2.98	-11.10, 0.71	0.08	0.11
Latino	-6.37	3.47	-13.20, 0.48	0.07	0.10
Asian	-3.43	4.70	-12.70, 5.84	0.47	0.66
**Working Memory**					
Age × Group	-0.10	0.05	-0.20, 0.00	0.04	0.10
Group, CRS	4.89	2.24	0.46, 9.31	0.03	0.09
Age	-0.02	0.03	-0.09, 0.05	0.57	0.57
BMI	0.00	0.06	-0.12, 0.12	0.98	0.98
Years of Education	0.29	0.14	0.01, 0.57	0.05	0.06
Sex, Male	0.95	0.84	-0.71, 2.60	0.26	0.49
African American	-1.55	0.91	-3.34, 0.25	0.09	0.11
Latino	-3.32	1.05	-5.40, -1.24	<0.01	<0.01
Asian	2.36	1.43	-0.46, 5.17	0.10	0.30
**Semantic Memory**					
Age × Group	-0.05	0.09	-0.23, 0.14	0.61	0.92
Group, CRS	-0.14	4.25	-8.53, 8.24	0.97	0.98
Age	-0.09	0.07	-0.22, 0.04	0.17	0.20
BMI	-0.01	0.11	-0.23, 0.21	0.91	0.98
Years of Education	0.86	0.27	0.33, 1.40	<0.01	<0.01
Sex, Male	1.43	1.62	-1.76, 4.62	0.38	0.49
African American	-4.24	1.73	-7.65, -0.83	0.02	0.03
Latino	-6.32	2.00	-10.30, -2.38	<0.01	<0.01
Asian	-4.63	2.70	-9.95, 0.69	0.09	0.30
**Visuospatial Abilities**					
Age × Group	-0.08	0.04	-0.15, 0.00	0.05	0.10
Group, CRS	2.49	1.71	-0.89, 5.86	0.15	0.29
Age	-0.05	0.03	-0.10, 0.00	0.05	0.10
BMI	0.06	0.04	-0.03, 0.14	0.22	0.44
Years of Education	0.29	0.11	0.07, 0.50	<0.01	0.02
Sex, Male	1.32	0.64	0.06, 2.58	0.04	0.24
African American	-2.81	0.69	-4.17, -1.44	<0.01	<0.01
Latino	-2.80	0.80	-4.38, -1.22	<0.01	<0.01
Asian	-1.46	1.09	-3.60, 0.69	0.18	0.36
**Episodic Memory**					
Age × Group	-0.12	0.05	-0.21, -0.03	0.01	0.05

Group, CRS	5.44	2.03	1.42, 9.45	0.01	0.05
Age	-0.05	0.03	-0.11, 0.01	0.11	0.16
BMI	-0.01	0.05	-0.12, 0.10	0.85	0.98
Years of Education	0.48	0.13	0.22, 0.73	<0.01	<0.01
Sex, Male	-0.63	0.76	-2.12, 0.87	0.41	0.49
African American	-0.47	0.82	-2.10, 1.15	0.57	0.57
Latino	-1.55	0.96	-3.44, 0.33	0.11	0.13
Asian	0.77	1.29	-1.78, 3.33	0.55	0.66

Group reference category is Control. Sex reference category is Female. Race reference category is White. FDR values were calculated using the Benjamini–Hochberg false-discovery rate procedure. Age × Group represents the interaction between age and CRS status.

Supplementary Table S4. Comparison of participants with and without available cytokine data

Variable	With cytokine data (n = 101)	Without (n = 77)
Age, mean ± SD	41.5 ± 15.4	41.1 ± 14.9
Male, n (%)	28 (27.7%)	22 (28.6%)
Education (years)	16.4 ± 2.8	16.3 ± 2.8
BMI	28.5 ± 5.4	28.1 ± 5.2
Asthma, n (%)	27 (26.7%)	33 (42.9%)
Atopy, n (%)	55 (54.5%)	37 (48.1%)
Nasal polyps among CRS, n (%)	25 (47.2%)	14 (51.9%)

Supplementary S3

Supplementary Methodology

This Supplementary document provides detailed information about the questionnaires, cognitive assessments, and venous blood sample collection for interleukin analysis.

Sleep and Sinonasal Outcome Questionnaires:

The first questionnaire was called the Pittsburgh Sleep Quality Index (PSQI). PSQI assesses sleep quality and disturbances over a one-month time interval. The questionnaire consists of 19 questions that measure seven components of sleep: subjective sleep quality, sleep latency, sleep duration, habitual sleep efficiency, sleep disturbances, use of sleep medication, and daytime dysfunction. The scores for each component are added to obtain a global PSQI score, which ranges from 0 to 21, with higher scores indicating poorer sleep quality [1].

We also used the Munich Chronotype Questionnaire (MCTQ), which measures sleep and rest time over a week. From this survey, we assessed variables such as the following SLAT-w: Sleep latency on workdays; SLAT-F: Sleep latency on free days; MSFSC: Mid-Sleep on Free days, sleep-corrected; these variables are indicative of sleep timing [2].

The next questionnaire was the Functional Outcomes of Sleep Questionnaire (FOSQ10). FOSQ measures the impact of sleep disturbance on a person's ability to function during

waking hours. It consists of 10 items that assess a person's ability to perform daily activities that may be affected by poor sleep quality, such as concentration, memory, and social functioning. The total score ranges from 0 to 40, with higher scores indicating better functional outcomes [3].
The next survey was the Epworth Sleepiness Scale (ESS). The ESS assesses the level of daytime sleepiness a person experiences. It consists of eight questions asking about the likelihood of dozing off or falling asleep in different situations, such as sitting and reading, watching television, or in a public place. Each question is scored on a scale of 0 to 3, with a maximum total score of 24. Scores of 10 or higher are generally considered to indicate excessive daytime sleepiness, which may be indicative of a sleep disorder or other underlying medical condition [4].
The fourth survey was the Sinonasal Outcome Test (SNOT-22). It assesses the health-related quality of life in patients with CRS. The questionnaire consists of 22 questions that cover various aspects of CRS, such as nasal symptoms, ear/facial symptoms, sleep, and emotional function. Each question has a score ranging from 0 to 5, with higher scores indicating more severe symptoms [5].
The fifth survey was the Chronic Sinusitis Survey. It assesses sinusitis-specific symptoms and medication use during the preceding 8 weeks. Each category was scored 0-4. The total scores were standardized to range from 0 to 100. In the case of CSS, lower total and subscale scores signify a greater impact of disease on the patient [6].

Cognitive Assessments:

Domain score construction: The cognitive battery consisted of 12 neuropsychological subtests grouped into 6 cognitive domains based on established neuropsychological assessment frameworks. Domain scores were calculated by summing the raw scores of the completed component subtests within each domain. Raw subtest scores were analyzed on their native measurement scales. Domain-level analyses used complete cases, meaning that a participant’s domain score was included when all component subtests for that domain were available. This approach was applied consistently with the complete-case statistical analysis described in Section 2.4 of the main manuscript.

**Cognitive domain**	**Component subtests used for domain score**
Episodic Memory	Word list immediate recall + delayed recall
Attention/Processing Speed	Digit Symbol Substitution + Number Comparison
Visuospatial Abilities	Progressive Matrices + Line Orientation
Semantic Memory	Boston Naming Test + Verbal Fluency + Category Fluency
Working Memory	Digit Span Forward + Digit Span Backward + Digit Span Ordering
Executive Function	Stroop Color + Stroop Reading + WTAR + NART

Episodic memory is assessed for the ability to learn, store, and retrieve information about unique personal experiences that occur in daily life [7]. A word list was shown to the participants, and they were asked to immediately and delayed recall the words.

To evaluate Attention/Processing Speed, the digit symbol substitution test and number comparison test were conducted. The digit symbol substitution test assessed processing speed and attention by requiring the individual to match a series of digits with corresponding symbols as quickly as possible. The number comparison test, on the other hand, evaluated the individual's ability to compare and evaluate numerical information by presenting pairs of numbers in various formats and orders [8].

Visuospatial Abilities were assessed using the Progressive Matrices and Line Orientation tests. The Progressive Matrices test measured nonverbal reasoning and spatial ability by presenting a series of matrices with missing pieces, and the individual was required to identify the missing piece that completes each matrix [9]. The Line Orientation test, on the other hand, evaluated the individual's spatial perception and visuospatial processing by presenting a series of lines at different angles, and the individual was required to identify the angle of each line relative to a reference line [10].

Boston Naming Test, Verbal Fluency, and Category Fluency tested semantic memory, specifically language processing. The Boston Naming Test is a language test that involves presenting pictures of objects to an individual and asking them to name the objects. The test is designed to assess the individual's ability to retrieve and produce words from memory and access and use semantic knowledge [11]. Verbal Fluency, also known as phonemic fluency, is a language test that involves asking the individual to generate as many words as possible within a specific category or starting with a specific letter in a given amount of time. The test is designed to assess the individual's ability to produce words from memory and to access and use semantic knowledge. The test is widely used in clinical and research settings to assess language function and executive function [12]. Category Fluency, also known as semantic fluency, is a language test that involves asking the individual to generate as many words as possible within a specific semantic category in each amount of time. The test is designed to assess the individual's ability to access and use semantic knowledge and to switch between categories [13].

The Digit Span Forward, Digit Span Backward, and Digit Span Ordering are three subtests of the Wechsler Adult Intelligence Scale (WAIS) and Wechsler Memory Scale (WMS), which are commonly used to test working memory. The Digit Span Forward test involves presenting a sequence of numbers to an individual and asking them to repeat the numbers back in the same order. The sequence's length is increased until the individual cannot recall the correct sequence. The Digit Span Backward test is like the Digit Span Forward test but requires the individual to repeat the sequence of numbers in reverse order. The Digit Span Ordering test is a variation of the Digit Span Backward test, where the individual is presented with a list of digits and asked to repeat them back in ascending order [8].

Stroop Color and Stroop Reading tests are cognitive assessment tools used to measure an individual's ability to inhibit interference from irrelevant stimuli. The Stroop Color test involves the presentation of color words (e.g., "red," "blue," "green") printed in different ink colors, and the individual is asked to name the ink color while ignoring the meaning of the word. The Stroop Reading test is a variation of the Stroop Color test where the

individual is presented with color patches and asked to read the name of the color while ignoring the color of the patch [14].
WTAR (Wechsler Adult Reading Test) and NART (National Adult Reading Test) are both tests of premorbid intelligence, which means that they are used to estimate an individual's intellectual functioning before any neurological damage or cognitive decline occurs [8]. These tests involve the individual reading aloud a list of words that are irregularly pronounced, and the score is based on the number of correctly pronounced words. The Stroop and reading tests both test executive function.

Interleukin Analysis:
Venipuncture was performed by trained phlebotomists using sterile techniques to minimize the risk of contamination. Upon collection, venous blood samples were transferred into Sodium Heparin tubes to prevent coagulation. The tubes were then centrifuged at 2150 rpm for 15 minutes to separate the plasma from cellular components. Plasma samples were carefully pipetted into labeled microcentrifuge tubes and stored at –80 degrees Celsius until further analysis.
References:

1. Buysse, D.J., et al., *The Pittsburgh Sleep Quality Index: a new instrument for psychiatric practice and research.* Psychiatry Res, 1989. **28**(2): p. 193-213.

2. Roenneberg, T., et al., *Epidemiology of the human circadian clock.* Sleep Med Rev, 2007. **11**(6): p. 429-38.

3. Weaver, T.E., et al., *An instrument to measure functional status outcomes for disorders of excessive sleepiness.* Sleep, 1997. **20**(10): p. 835-43.

4. Johns, M.W., *A new method for measuring daytime sleepiness: the Epworth sleepiness scale.* Sleep, 1991. **14**(6): p. 540-5.

5. Hopkins, C., et al., *Psychometric validity of the 22-item Sinonasal Outcome Test.* Clin Otolaryngol, 2009. **34**(5): p. 447-54.

6. Quintanilla-Dieck, L., et al., *Comparison of disease-specific quality-of-life instruments in the assessment of chronic rhinosinusitis.* Int Forum Allergy Rhinol, 2012. **2**(6): p. 437-43.

7. Dickerson, B.C. and H. Eichenbaum, *The episodic memory system: neurocircuitry and disorders.* Neuropsychopharmacology, 2010. **35**(1): p. 86-104.

8. Wechsler, D., *Wechsler adult intelligence scale, 3rd edn San Antonio.* TX: The Psychological Corporation, 1997.

9. Raven, J.C., *Guide to the standard progressive matrices, sets A, B, C, D and E.* (No Title), 1960.

10. Ekstrom, R.B. and H.H. Harman, *Manual for kit of factor-referenced cognitive tests, 1976*. 1976: Educational testing service.

11. Goodglass, H., E. Kaplan, and S. Weintraub, *Boston naming test*. 1983: Lea & Febiger Philadelphia, PA.

12. Henry, J.D. and J.R. Crawford, *Verbal fluency deficits in Parkinson's disease: a meta-analysis.* Journal of the International Neuropsychological Society, 2004. **10**(4): p. 608-622.

13. Reverberi, C., et al., *Semantic fluency: cognitive basis and diagnostic performance in focal dementias and Alzheimer's disease.* Cortex, 2014. **54**: p. 150-64.

14. Golden, C., S.M. Freshwater, and Z. Golden, *Stroop color and word test.* 1978.
